# A CT-based transfer learning approach to predict NSCLC recurrence: The added-value of peritumoral region

**DOI:** 10.1371/journal.pone.0285188

**Published:** 2023-05-02

**Authors:** Samantha Bove, Annarita Fanizzi, Federico Fadda, Maria Colomba Comes, Annamaria Catino, Angelo Cirillo, Cristian Cristofaro, Michele Montrone, Annalisa Nardone, Pamela Pizzutilo, Antonio Tufaro, Domenico Galetta, Raffaella Massafra

**Affiliations:** I.R.C.C.S. Istituto Tumori “Giovanni Paolo II”, Bari, Italy; Harvard Medical School, UNITED STATES

## Abstract

Non-small cell lung cancer (NSCLC) represents 85% of all new lung cancer diagnoses and presents a high recurrence rate after surgery. Thus, an accurate prediction of recurrence risk in NSCLC patients at diagnosis could be essential to designate risk patients to more aggressive medical treatments. In this manuscript, we apply a transfer learning approach to predict recurrence in NSCLC patients, exploiting only data acquired during its screening phase. Particularly, we used a public radiogenomic dataset of NSCLC patients having a primary tumor CT image and clinical information. Starting from the CT slice containing the tumor with maximum area, we considered three different dilatation sizes to identify three Regions of Interest (ROIs): CROP (without dilation), CROP 10 and CROP 20. Then, from each ROI, we extracted radiomic features by means of different pre-trained CNNs. The latter have been combined with clinical information; thus, we trained a Support Vector Machine classifier to predict the NSCLC recurrence. The classification performances of the devised models were finally evaluated on both the hold-out training and hold-out test sets, in which the original sample has been previously divided. The experimental results showed that the model obtained analyzing CROP 20 images, which are the ROIs containing more peritumoral area, achieved the best performances on both the hold-out training set, with an AUC of 0.73, an Accuracy of 0.61, a Sensitivity of 0.63, and a Specificity of 0.60, and on the hold-out test set, with an AUC value of 0.83, an Accuracy value of 0.79, a Sensitivity value of 0.80, and a Specificity value of 0.78. The proposed model represents a promising procedure for early predicting recurrence risk in NSCLC patients.

## Introduction

Lung cancer is one of the most aggressive cancer types with a 5-year relative survival rate of only 19%. Non-small cell lung cancer (NSCLC) accounts for 85% of lung cancer cases and is one of the most fatal cancers worldwide [1]. Treatment approaches for NSCLC patients differ depending on stage, histology, genetic alterations, and patient’s condition. Locally advanced NSCLC patients are non-surgical candidates and currently treated with chemoradiotherapy eventually followed by immunotherapy. On the other hand, for early stages of NSCLC, surgically resection and consequent adjuvant chemotherapy are recommended. Though surgically resection remains the only potentially curative treatment for early-stage NSCLC, 30–55% of these patients develop a post-resection tumor recurrence within the first 5 years. Several studies demonstrated patients’ outcome after surgically resection is often affected by an underestimation of the tumor stage, due to the presence of occult micro-metastatic cancer cells undetectable by standard staging methods, such as modern diagnostic imaging. Also, in some cases, surgery itself could lead to the dissemination of cancer cells [2]. Thus, an early identification of which patients are more prone to develop a NSCLC recurrence is crucial to define personalized treatment approaches and improving patients’ prognosis.

Actually, the application of artificial intelligence techniques could be fundamental in developing tools able to support clinicians in defining personalized therapeutic surveillance plans, after identifying patients at high risk of relapse.

In the clinical pathway, biomedical imaging, such as magnetic resonance (MR), computed tomography (CT), or positron emission tomography (PET), plays a pivotal role, offering several non-invasive modalities for the high-resolution three-dimensional visualization and characterization of the cancer lesion. Besides, the predictive and prognostic power of radiomic signature extracted from biomedical images is now well established in the scientific community [3–8]. So far, several radiomic-based models have been proposed for lung cancer setting to solve different tasks [9–13]. While several works are focused on the prediction of histological outcome, tumor staging, recurrence free survival and overall survival for NSCLC patients [14–32], the state of the art is poor of models designed for early prediction of disease recurrence [33–41]. Additionally, even though all the proposed models show encouraging results, they are not yet suitable for a clinical application, even when they involve genomic-based models that are expensive and time-consuming procedures [33]. Therefore, the early prediction of recurrence in NSCLC patients is still an unmet clinical need with a strong translational interest.

To this end, herein we propose a radiomic-based model for predicting the NSCLC recurrence exploiting features extracted from pre-treatment CT images throughout pre-trained Convolutional Neural Networks (CNNs). Pre-trained CNNs refer to a transfer learning approach which allows to extract radiomic features from images according to which the networks have previously learned during training on a very huge (millions) number of images of different nature. Thus, the knowledge acquired from the network during this training phase, such as dots and edges, as well as high-level features like shapes and objects from raw images, has been then transferred and applied on CT images of our sample patients [10, 42–46]. For our purpose, we used a public database contained both CT images and clinical data of NCSLC patients, and we analyzed them conjointly to develop a suitable supervised machine learning model [47]. Specifically, we compared the results obtained using multiple state-of-the-art pre-trained CNNs for radiomic feature extraction, and we evaluated performances achieved examining different regions of interest (ROIs) at different dilatations, to investigate the predictive power of the peritumoral region, namely, the tissue connecting the tumor and the normal tissue.

This manuscript is organized as follows: in Section 2, Materials and Methods, we introduce the used dataset, the feature extraction procedure by a transfer learning approach, and the designed learning model; in Section 3–4, Results and Discussion, we present and discuss the computed performances comparing our study with the state-of-the-art about NSCLC recurrence prediction.

## Materials and methods

### Experimental dataset

In this work, we used a public radiogenomics dataset of NSCLC available in the Cancer Imaging Archive (TCIA) [47]. Both imaging and clinical data have been de-identified by TCIA and approved by the Institutional Review Board of the TCIA hosting institution. Ethical approval was reviewed and approved by Washington University Institutional Review Board protocols. Written informed consent was obtained from all individual participants involved.

The whole database consisted of 211 subjects divided in two cohorts:

1. the R01 cohort comprising 162 patients (38 females and 124 males, age at scan: mean 68, range: 42–86) from Stanford University School of Medicine (69) and Palo Alto Veterans Affairs Healthcare System (93) recruited between April 7^th^ 2008 and September 15^th^, 2012;
2. the second AMC cohort consists of 49 additional subjects (33 females, 16 males, age at scan: mean 67, range 24–80) was retrospectively collected from Stanford University School of Medicine based on the same criteria.

Since only (1) the database 1 included the segmentations of the axial CT images, for this preliminary study we focused on the cohort R01. Besides, since the tumor segmentation masks was not available for 18 patients belonged to the cohort R01, the final number of patients involved in this study was equal to 144, of which 40 (27.78%) with a recurrence event within 8 years from the first tumor diagnosis. For each patient, a CT image in DICOM format, as well as clinical data were provided. Concerning CT images, these were acquired by preoperative CT scans with a thickness of 0.625–3 mm and an X-ray tube current at 124–699 mA at 80–140 KVp. Consequently, the related segmentations were defined on the axial CT image series by thoracic radiologists with more than 5 years of experience and adjusted using ePAD software [47].

For each patient, along with the CT image, the following clinical data were collected: age at diagnosis, weight, gender (values: female, male), pack Years, histology (values: adenocarcinoma, squamous cell carcinoma, not otherwise specified), pathological T stage (values: T1, T2, T3, T4) [48], pathological N stage (values: N0, N1, N2) [48], histopathological grade (values: G1, G2 and G3) [49], lymph-vascular invasion (values: absent, present, not collected) and pleural invasion (values: yes, no). An overview about the sample properties is provided by Table 1.

**Table 1:** Clinical features distribution over the study population.

Feature	Distribution
**Overall**	144; 100%
**Age at diagnosis**	
Median; [q_1_, q_3_]	69; [64, 76]
**Weight (Ibs)**	
Median; [q_1_, q_3_]	173.5; [145.13, 198.90]
Nan (abs; %)	10; 6.94%
**Gender**	
Female (abs; %)	36; 25%
Male (abs; %)	108; 75%
**Pack Years**	
Median; [q_1_, q_3_]	40; [20, 54]
Nan (abs; %)	27; 18.75%
**Histology**	
Adenocarcinoma (abs; %)	112; 77.77%
Squamous cell carcinoma (abs; %)	29; 20.14%
Not otherwise specified (abs; %)	3; 2.08%
**Pathological T stage**	
T1 (abs; %)	74; 51.39%
T2 (abs; %)	49; 34.03%
T3 (abs; %)	16; 11.11%
T4 (abs; %)	5; 3.47%
**Pathological N stage**	
N0 (abs; %)	115; 79.86%
N1 (abs; %)	12; 8.33%
N2 (abs; %)	17; 11.8%
**Histopathological Grade**	
G1 (abs; %)	37; 25.69%
G2 (abs; %)	80; 55.56%
G3 Poorly differentiated (abs; %)	27; 18.75%
**Lymph-vascular invasion**	
Absent (abs; %)	121; 84.03%
Present (abs; %)	18; 12.5%
Nan (abs; %)	5; 3.47%
**Pleural invasion**	
No (abs; %)	105; 72.92%
Yes (abs; %)	39; 27.08%

“Nan” stands for “Not A Number”, “abs” stands for “absolute value”.

### Feature extraction by transfer learning approach

For each patient, the first step consisted in automatically identifying, among all segmentation masks, the mask with largest tumor area, that is, the segmentation mask characterized by the greatest number of pixels having an intensity value equals to 255, i.e., white pixels. Segmentation masks, which were generated by authors of the public database, were obtained using an unpublished automatic segmentation algorithm based on semantic annotations ascribed by an expert radiologist, and then reviewed by two thoracic radiologists with more than 5 years of experience which edited them as necessary [47].

**Fig 1:**
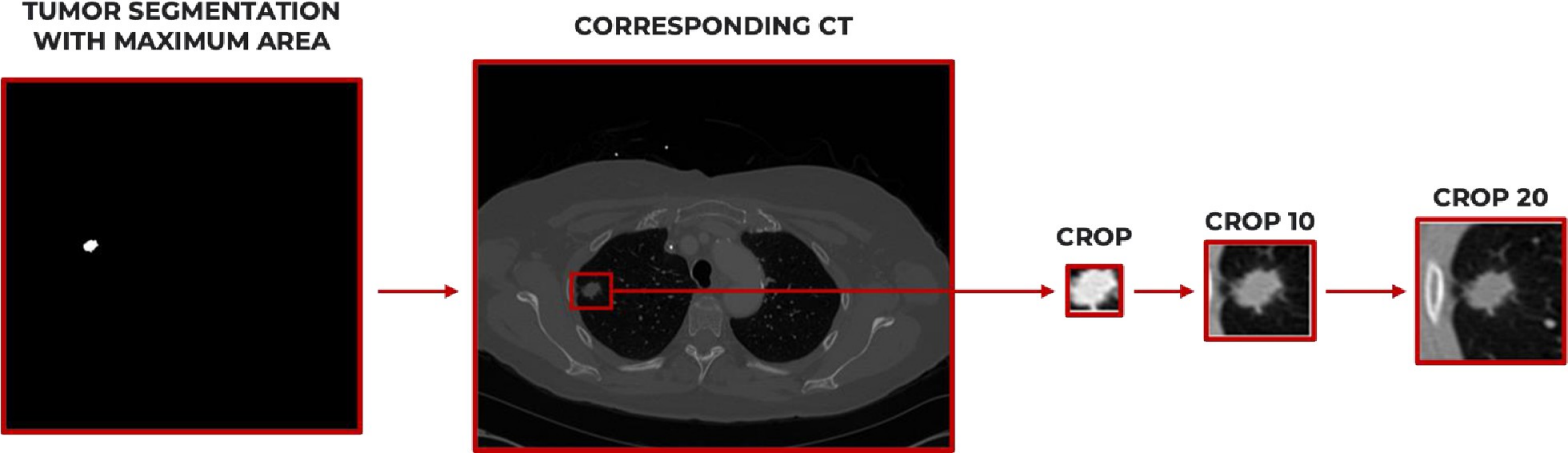
ROI extraction process. After identifying the tumor segmentation with maximum area, along with the corresponding CT image, three ROIs were extracted for each patient: CROP, CROP 10 and CROP 20.

After identifying the corresponding CT slice, we defined a bounding box around the extremal points of the tumour in the four planar x-y dimensions. So, we cropped the correspondent CT slide considering three different dilatation sizes: 0 (no dilatations), 10 and 20 additional pixels along the four extremal points. In this way, for each patient, we identified the following Regions of Interest (ROI)s: CROP (with no dilations), CROP 10 (obtained adding 10 pixels) and CROP 20 (obtained adding 20 pixels). The whole ROI extraction procedure is depicted in Fig 1.

**Fig 2:**
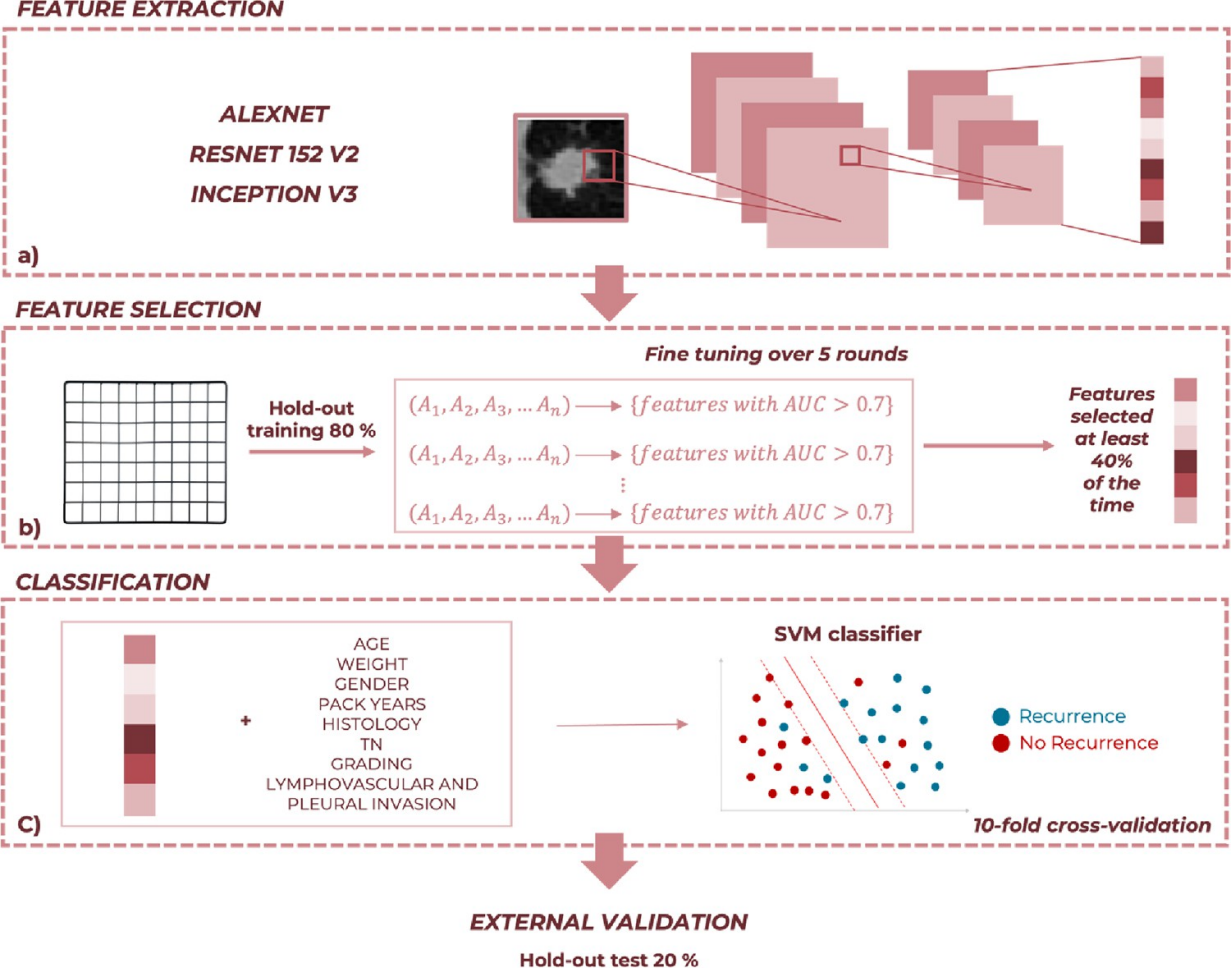
Schematic overview of the proposed approach. (a) After extracting radiomic features by means of three different pre-trained CNNs from each identified crop, (b) we performed a feature selection procedure within a 10-fold cross-validation scheme over 5 rounds on the hold-out training set. (c) Then, we trained a SVM classifier on the hold-out training set exploiting both clinical data and radiomic features extracted in the previous step. Finally, we performed an external validation on the hold-out test set.

Next, as depicted in Fig 2A, from each ROI we extracted radiomic features using three pre-trained convolutional neural networks (CNNs), namely, AlexNET, ResNet152V2 and InceptionV3, after resizing all ROIs to the specific dimension required by each network. Pre-trained CNNs have been trained on more than a million images belonging to a subset of the ImageNet database [50], and can classify images into 1000 object categories. Pre-trained networks are mainly characterized for their accuracy and their relative running time. Therefore, choosing the pre-trained CNN to be implemented means finding a well-balanced compromise between these characteristics. Accordingly, pre-trained CNNs we selected represent three different well-balanced compromises between accuracy and relative running time [51].

Concerning AlexNET [44], which requires input images resized to 227×227 pixels, we extracted features from the pool2 layer of the network architecture which corresponds to the second pooling layer after the second convolutional layer of the network. The pool2 layer has an output with dimensions of 13×13×256 that is flattening to a single 43264-length vector. As consequence, the number of extracted features is 43264 in total for each ROI of every patient.

Concerning ResNet152V2 [52], which requires input images resized to 224×224 pixels, we extracted features using the *max_pooling2d layer*, which corresponds also in this case to the second pooling layer after the second convolutional layer and has an output with dimensions of 28×28×256 flattened to a single 200794-length vector. Thus, for each ROI of every patient the number of features extracted is equal to 200794.

Finally, we extracted features from the *max_pooling2d layer*, the second one after the second convolutional layer of the InceptionV3 network architecture [53], after resizing images to 299×299 pixels. The max_pooling2d layer has an output with dimensions of 35×35×192 that is flattening to a single 235200-length vector. As consequence, the number of extracted features is 235200 in total for each ROI of every patient.

So, for each pre-trained network, we exploited pool2 layer for feature extraction. This is because pool2 layer is one of the initial layers of the network and returns low-level features, i.e., representations of local details of an image, such as edges, dots, and curves. These details would otherwise be obscured considering only global information extracted from later layers of the network. Additionally, we extracted features from a pooling layer rather than a convolutional layer to preserve the invariance to truncation, occlusion, and translation [54].

All the analysis steps have been performed by using MATLAB R2022a (Mathworks, Inc. Natick, MA, USA) software.

### Learning model

Using both clinical data and radiomic features extracted in the previous step, our aim was to devise a model for predicting recurrence event in NCSLC. The flowchart of the implemented method is shown in Fig 2. After implementing the feature extraction procedure previously described, we performed a stratified randomly sampling on the overall dataset, in order to split the 144 NSCLC patients in a hold-out training set, containing 80% of the sample, and a hold-out test set, containing 20% of the sample. As a consequence, the hold-out training set consisted of 116 patients, of which 81 control cases and 35 recurrence cases. While the hold-out test set consisted of 28 patients, of which 23 control cases and 5 recurrences.

Consequently, we developed nine learning models which discriminate between recurrence and non-recurrence patients, exploiting normalized features extracted by means of the three different pre-trained CNNs from the CROP, the CROP 10 and the CROP 20, by turns.

For each devised model, we firstly selected the only features whose variance was not equal to zero, and then we performed a feature selection procedure on the hold-out training set (Fig 2B). Thus, we recorded the features with an Area Under the Curve (AUC) value greater than 0.7 over 5 rounds of a finetuning procedure. Specifically, for each round, the hold-out training set was partitioned into 10 smaller sets, and each of these sets was removed by turns for evaluating features predictive power.

At the end of this iterative procedure, we selected the subset of radiomic features that showed an AUC above this threshold at least 40% for AlexNET, 60% for ResNet152V2 and 100% InceptionV3. These thresholds have been found to be the optimal ones after evaluating classification performances achieved by our model according to all possible frequencies. Though these frequencies differ from each other due to the different architectures of the employed networks, they represent the best trade-off between high performances and low-dimensional datasets. Interim results were not reported to not burden the discussion.

According to this features reduction step, we obtained a subset of significative features for each applied CNN. Then, after estimating the missing clinical data of the database by means of the Miss Forest imputation technique [55], we combined each radiomic feature subset with the clinical data, in order to train a SVM classifier on the hold-out training set within a 10-fold cross-validation scheme over 5 rounds, as depicted in Fig 2C. SVM is a supervised machine learning model which detects the hyperplane that has the maximum distance between data points of both classes, through a specific kernel function. For our study the linear function was adopted. Finally, we evaluated all the developed classification models on the hold-out test set using the optimal feature subset identified on hold-out training set (external validation in Fig 2).

For both the hold-out training and the hold-out test set we evaluated performances of all used models in terms of AUC, as well as Accuracy (Acc), Sensitivity (Sens), Specificity (Spe), which are metrics calculated by identifying the optimal threshold by means of a Youden’s index test [56].

## Results

Table 1 summarized the characteristics of the analyzed sample. For Age at Histological Diagnosis, Weight, and Pack Years median, first quartile q_1_ and third quartile q_3_ are reported. For the other clinical features, the absolute and relative frequencies are reported.

Classification performances achieved by all models on CROP, CROP 10 and CROP 20 images are summarized in Tables 2–4, respectively. Specifically, each table includes performances obtained on both the hold-out training and the hold-out test sets, along with the number of radiomic features selected within the feature selection procedure and exploited for training the related model.

**Table 2:** Classification performances achieved with CROP images on both hold-out training and hold-out test sets.

		HOLD-OUT TRAINING				HOLD-OUT TEST			
	CNN features	AUC	Acc	Sens	Spe	AUC	Acc	Sens	Spe
**AlexNET+clinical**	8	0.73	0.61	0.63	0.60	0.64	0.71	0.40	0.78
**ResNet152V2+clinical**	16	0.71	0.66	0.71	0.63	0.54	0.39	1.0	0.26
**InceptionV3+clinical**	27	0.66	0.67	0.74	0.64	0.68	0.68	0.80	0.65

**Table 3:** Classification performances achieved with CROP 10 images on both hold-out training and hold-out test sets.

		HOLD-OUT TRAINING				HOLD-OUT TEST			
	CNN features	AUC	Acc	Sens	Spe	AUC	Acc	Sens	Spe
**AlexNET+clinical**	4	0.73	0.61	0.63	0.60	0.79	0.82	0.80	0.83
**ResNet152V2+clinical**	11	0.80	0.78	0.66	0.84	0.54	0.50	1.0	0.39
**InceptionV3+clinical**	11	0.75	0.70	0.83	0.64	0.72	0.89	0.60	0.96

**Table 4:** Classification performances achieved with CROP 20 images on both hold-out training and hold-out test sets.

		HOLD-OUT TRAINING				HOLD-OUT TEST			
	CNN features	AUC	Acc	Sens	Spe	AUC	Acc	Sens	Spe
**AlexNET+clinical**	7	0.73	0.61	0.63	0.60	0.83	0.79	0.80	0.78
**ResNet152V2+clinical**	17	0.78	0.72	0.83	0.68	0.63	0.79	0.60	0.83
**InceptionV3+clinical**	6	0.72	0.63	0.86	0.53	0.73	0.68	1.0	0.61

Concerning CROP images, Table 2 shows how the best performances on the hold-out training set were reached with 8 residual radiomic features extracted by AlexNET: AUC = 0.73, Acc = 0.61, Sens = 0.63, and Spe = 0.60. On the other hand, the best performances on the hold-out test set were obtained involving 27 residual features extracted by InceptionV3: AUC = 0.68, Acc = 0.68, Sens = 0.80, and Spe = 0.65.

Considering CROP 10 images, Table 3 reveals how the best performances on the hold-out training set have been reached exploiting 11 residual radiomic features extracted by ResNet152V2: AUC = 0.80, Acc = 0.78, Sens = 0.66, and Spe = 0.84. However, on the hold-out test set, the best performances were obtained by analyzing 4 residual radiomic features extracted via AlexNET: AUC = 0.79, Acc = 0.82, Sens = 0.80, and Spe = 0.83. referring to InceptionV3, its performances were stable on both the hold-out training and hold-out test sets.

Finally, as far as CROP 20 images, Table 4 shows how the best performances on the hold-out training set have been achieved involving 17 residual radiomic features extracted by ResNet152V2: AUC = 0.78, Acc = 0.72, Sens = 0.83, and Spe = 0.68. These performances decreased on the hold-out test set in terms of Sensitivity (0.60). Actually, the best performances on the hold-out test set were reached with 7 residual radiomic features extracted by AlexNET: AUC = 0.83, Acc = 0.79, Sens = 0.80, and Spe = 0.78.

Comparing results obtained on the hold-out test set analyzing the three different CROPs, performances achieved on CROP 20 images resulted the best ones.

Actually, for each patient, further ROIs were identified exploring other dilatation sizes, such as, 30, 40, 50 and 60 additional pixels along the four extremal points (S1 Fig). However, classification performances achieved by our models on all these images decreased significantly, probably because of a too large zone of peritumoral tissue considered which could also include surrounding regions, such as, the backbone, which could be confounding elements for model learning. With the purpose of showing how surrounding regions containing confounding elements really affect performances, in S2 Fig we depicted AUC values achieved by models trained on features extracted by AlexNET pool2 layer from CROP 0, CROP 10, CROP 20, CROP 30, CROP 40, CROP 50 and CROP 60. Moreover, middle dilatation sizes were investigated, but the most appropriate criterion resulted the one we adopted. Interim results were not reported to not burden the discussion.

Finally, pool1, pool2 and pool5 layers of the AlexNET network were exploited to extract features on the CROP 20 images, with the aim of demonstrating the second *pooling layer* provides the most relevant information (S3 Fig).

## Discussion

An early and accurate prediction of recurrence risk in NSCLC patients during diagnosis could be essential to promptly designate risk patients to more aggressive medical therapies, and, on the other hand, to spare no risk patients from unnecessary invasive treatments [1]. For this purpose, it could be important to design a model able to assess in NSCLC patients the recurrence risk during diagnosis. Nowadays, in the clinical practice, CT imaging represents the gold standard for NSCLC diagnosis. Therefore, the goal of this study is to define a model able to predict the NSCLC recurrence risk exploiting both clinical data and a CT image of the primary tumor, which are both acquired during the screening phase.

We analyzed a public radiogenomic database, from which a sub-cohort of 144 patients with available CT images, segmentation tumor masks and clinical data have been selected [47]. In order to evaluate the information contained both in the tumor region and in the peritumoral area, once the image with largest tumor was identified, we cropped the image with dilatation sizes 0, 10 and 20 and extracted radiomic features via CNNs. The entire sub-cohort was divided into a hold-out training dataset and a hold-out test dataset corresponding to the 80% and 20% of the entire sample, respectively. Then, after reducing the radiomic features and combining them with clinical information a linear SVM classifier was trained and the performances on the hold-out training set and the hold-out test set were computed.

We have explored various CNNs, namely, AlexNET, ResNET152V2, and InceptionV3, and then we compared the related performances after suitably reducing the extracted features. Our best results were obtained investigating the predictive power of CROP 20 images, which are the images containing more peritumoral area. Particularly, on the hold-out training set our model achieved an AUC value equals to 0.73, an Accuracy equals to 0.61, a Sensitivity equals to 0.63, and a Specificity equals to 0.60. Even more promising performances were achieved on the hold-out test set with an of AUC 0.83, an Accuracy of 0.79, a Sensitivity of 0.80, and a Specificity 0.78. These results represent the best performances in terms of balance between hold-out training and hold-out test sets. While ResNET152V2 and InceptionV3 seem to be generally more performing on the hold-out training set, AlexNET appeared to give better performances on the independent test. Hence, classification performances resulted partially sensitive to pre-trained CNN choice due to the different accuracy characterizing pre-trained networks. Indeed, choosing a pre-trained CNN to be implemented means finding a well-balanced compromise between accuracy and relative running time.

Moreover, comparing these results with the ones obtained by analyzing both images without dilatations (CROP) and images containing a smaller dilatation (CROP 10), it is evident how the peritumoral region allowed us to retrieve more discriminant information about NSCLC recurrence prediction. As previously reported by our group in a study assessing the sentinel lymph-node status in breast cancer patients by ultrasound images of the primary tumor, we concluded the peritumoral region was essential for accurate predicting the outcome [6]. Other dilatation sizes, such as, 30, 40, 50 and 60 additional pixels, as well as middle dilatation sizes, were also investigated. On the one hand, classification performances achieved on CROP 30, CROP 40, CROP 50 and CROP 60 images decreased significantly, probably because of a too large zone of peritumoral tissue considered which could also include surrounding regions, such as, the backbone, which could be confounding elements for model learning. On the other hand, middle dilatation sizes did not appreciably contribute to improve classification performances. Consequently, the most appropriate criterion resulted the one we adopted.

Our results are comparable with those obtained by Wang et al. who analyzed CT images from a cohort of 157 NSCLC patients using only handcrafted-radiomic features, which are however operator dependent. In their study, they reached an Accuracy equals to 0.85 [37].

On the other hand, S. Hindocha et al. developed a model able to predict recurrence, recurrence-free survival, and overall survival of NSCLC patients, by employing only clinical features collected from a cohort of 657 patients. Considering the recurrence prediction, authors reached an AUC value equals to 0.69 and 0.72 for the validation and external datasets, respectively [38].

With respect to NSCLC recurrence studies involving features extracted by means of convolutional neural networks, P. Aonpong et al. used the same radiogenomic database analyzed in the present study to predict the NSCLC recurrence devising a genotype-guided radiomic model [33]. For their specific goal, a sub-cohort of 88 patients was considered. Their model predicted the NSCLC recurrence via gene expression data extracted from CT images vis CNNs and achieved an AUC of 0.77, and Accuracy of 0.83, a Sensitivity of 0.95, and a Specificity of 0.59.

Besides, G. Kim et al. recently proposed an ensemble-based prediction model for NSCLC recurrence involving 326 patients also including our dataset. They developed three neural network models trained combining clinical data, such as tumor node stage, handcrafted radiomic features, and deep learning radiomic features [35]. The final performances of clinical, handcrafted and deep-learned features together were AUC equal to 0.77, Sensitivity equals to 0.80, and Specificity equals to 0.73.

The best performances obtained in our study have been compared with those available in the literature, to the best of our knowledge, (Table 5).

**Table 5:** NSCLC recurrence prediction: A comparison among performances achieved by the state-of-the-art models.

	N. of patients	Model	Performances
Wang et al. (2019) [37]	157	Handcrafted Radiomic features based	Acc = 0. 85
Aonpong et al. (2021) [33]	88	CNN + gene-expression based	AUC = 0.77
			Acc = 0.83
Kim et al. (2022) [35]	326	CNN based + Handcrafted Radiomic based + Clinical based	AUC = 0.77
			Acc = 0.73
Hindocha et al. (2022) [38]	657	Clinical based	AUC = 0.69
Best proposed model	144	AlexNET CNN + clinical based	AUC = **0.83**; Acc = **0.79**

Accordingly, compared to the main state-of-the-art, our proposal shows better performing results, except with reference to models using genomic information. In this regard, in our study we aimed to devise a model to predict the NSCLC recurrence, purposely neglecting the genomic information provided by the clinical features EGFR and KRAS, that are clinically expansive and time-consuming to obtain. Furthermore, even though studies for predicting NSCLC recurrence involving both deep and clinical features already exist [33, 35], the original aspect of our study is the analysis of CT images with different dilatation (crops) levels and different CNNs. Using a different CNN, as well as analyzing a different dilatation level, can affect the final performances of the model. In fact, our results were extremely influenced by the thickness of peritumoral region considered, and our best performances were obtained investigating the predictive power of CROP 20 images. As well, though we exploited three pre-trained CNNs characterized by a well-balance compromise between accuracy and relative running time, performances were also influenced by network accuracy. Thus, in our future work, we will also investigate the predictive power of other pre-trained networks, such as DenseNET and Vision Transformer, as well as end-to-end models developed training CNNs on a more conspicuous data sample.

Besides, other limitations of our study deal with its retrospective design and the limited dimension of the dataset. With a larger dataset, it could be possible to achieve higher performances and improve the model. For this purpose, in our future work we will collect a private database of NSCLC patients, also including more histopathological features of the primary tumor, along with CT images acquired during the screening phase.

## Conclusion

The current study proposes an artificial intelligence-based model for early predicting recurrence risk in patients affected by NSCLC exploiting only data acquired during diagnosis, namely, clinical variables and a primary tumor CT image. Specifically, in this study we investigated the discriminant power of different CNNS employed for automatically extracted radiomics features from three different regions of interest, identified considering different thickness of peritumoral region. Despite the promising results achieved by our model analyzing the ROI containing the maximum peritumoral area, for our future work we aim to collect a private database of NSCLC patients, including both histopathological features and a CT image of the primary tumor. Moreover, it could be interesting to include the use of the *Explainable Artificial Intelligence* that through the years has gained a lot of attention in order to overcome the “black-box” nature of artificial intelligence algorithms, trying to better understand and explain the choices made by these models [57].

## Supporting information

S1 Fig Visualization of all ROIs extracted from each patient. After identifying the tumor segmentation with maximum area, along with the corresponding CT image, six ROIs were initially extracted for each patient, in addition to the CROP without dilation: CROP 10, CROP 20, CROP 30. CROP 40, CROP 50 and CROP 60.
(TIFF)

S2 Fig AUC values achieved by models trained on features extracted by AlexNET pool2 layer from CROP 0, CROP 10, CROP 20, CROP 30, CROP 40, CROP 50 and CROP 60. (TIFF)

S3 Fig Radar chart depicting classification performances achieved exploiting features extracted by pool1, pool2 and pool5 layers of the AlexNET network on the CROP 20 images. Performances achieved exploiting features extracted from pool2 layer resulted the best one.
(TIFF)
