# Supplementary figures and images for: A CT-based transfer learning approach to predict NSCLC recurrence: The added-value of peritumoral region
Source: PLoS One. 2023 May 2;18(5):e0285188. doi: 10.1371/journal.pone.0285188 (PMC10153708; doi:10.1371/journal.pone.0285188)

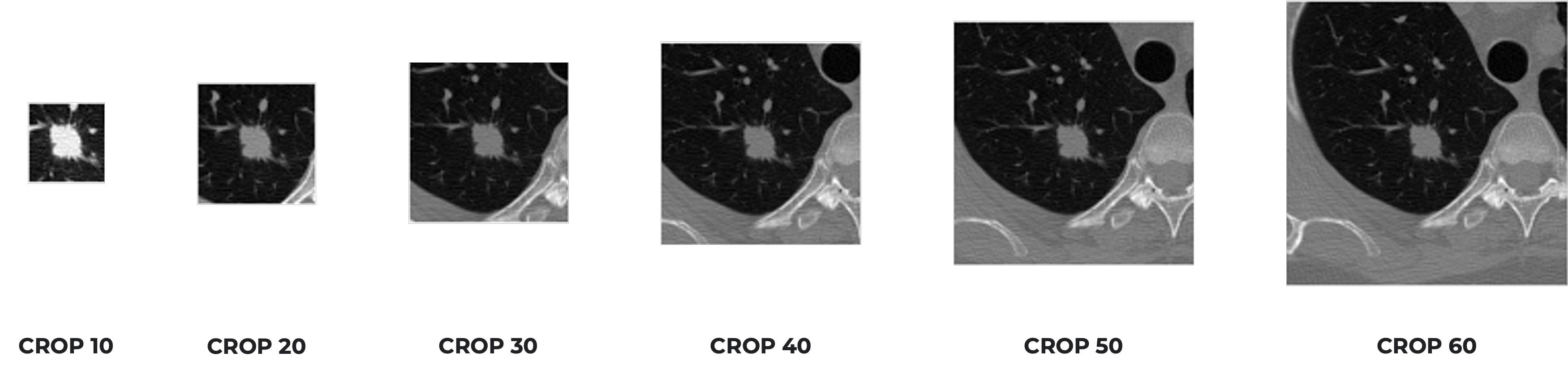

Supplement: S1 Fig — After identifying the tumor segmentation with maximum area, along with the corresponding CT image, six ROIs were initially extracted for each patient, in addition to the CROP without dilation: CROP 10, CROP 20, CROP 30. CROP 40, CROP 50 and CROP 60. (TIFF) [file pone.0285188.s001.tiff]

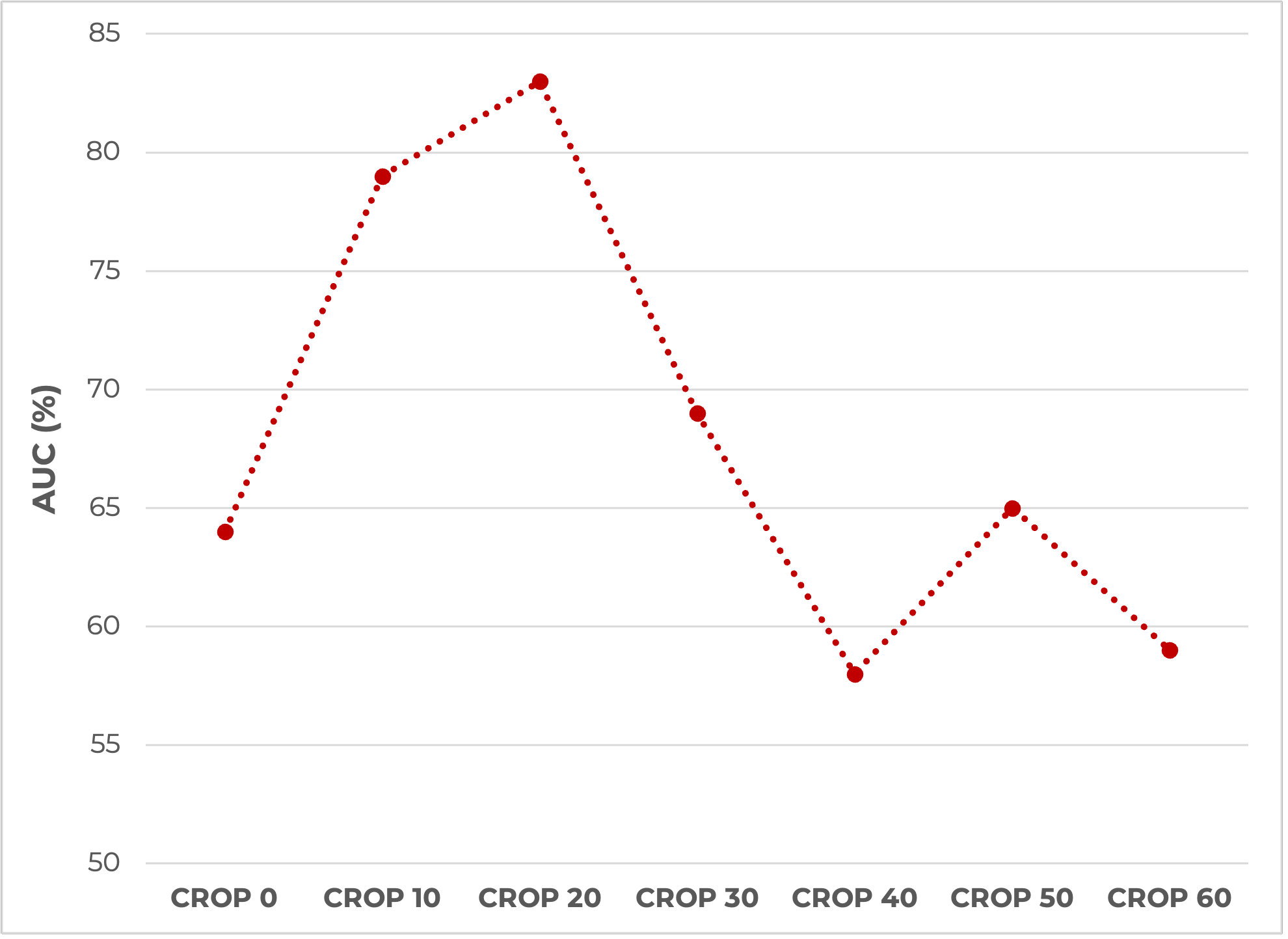

Supplement: S2 Fig — (TIFF) [file pone.0285188.s002.tiff]

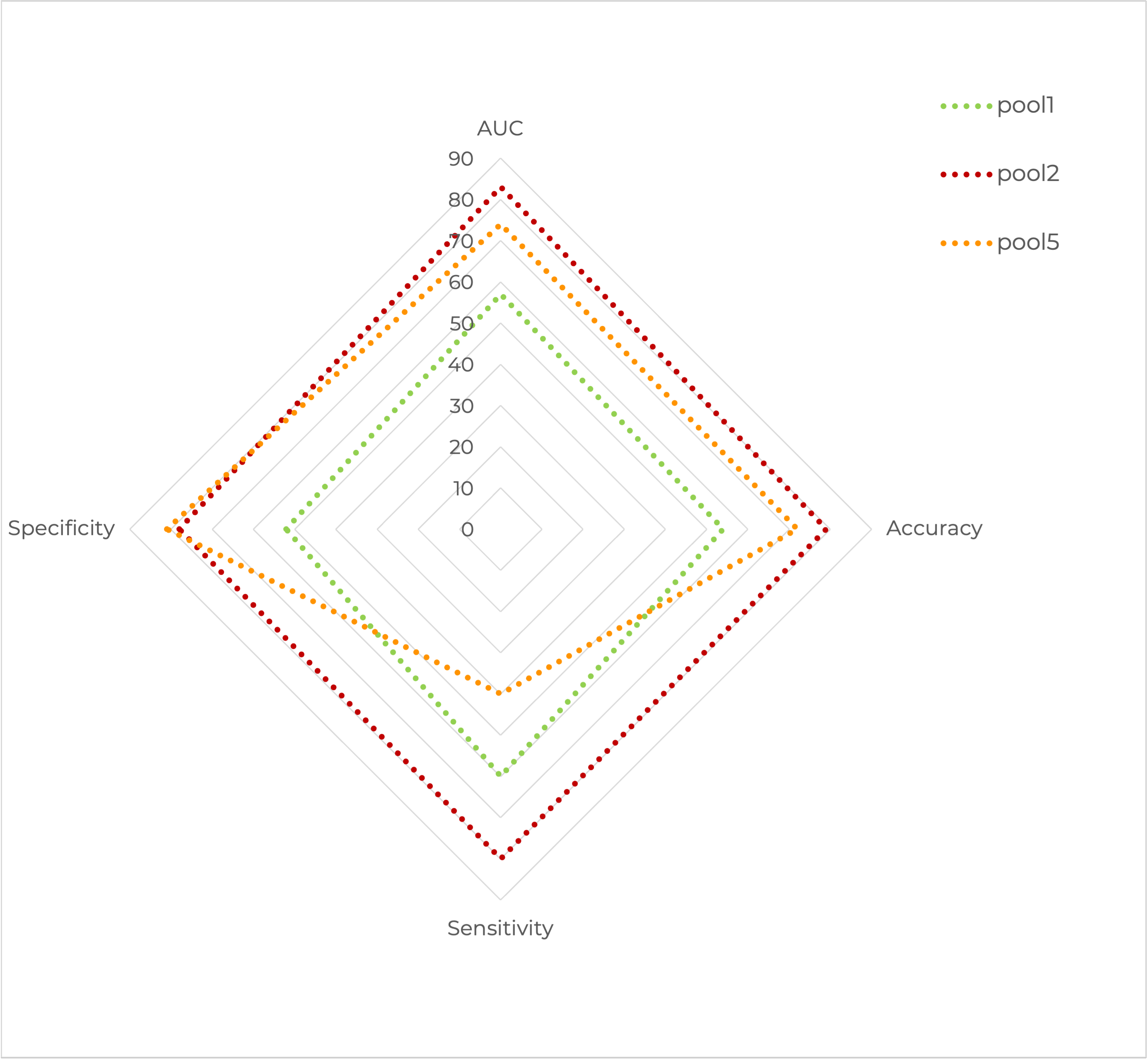

Supplement: S3 Fig — Performances achieved exploiting features extracted from pool2 layer resulted the best one. (TIFF) [file pone.0285188.s003.tiff]
